# Improving yields by switching central metal ions in porphyrazine-catalyzed oxidation of glucose into value-added organic acids with SnO_2_ in aqueous solution

**DOI:** 10.3389/fchem.2023.1114454

**Published:** 2023-05-30

**Authors:** Quanquan Zhang, Xin Li, Xingwang Wang, Xin Huang, Yuncai Liu, Fengshou Wu, Bingguang Zhang, Kejian Deng

**Affiliations:** ^1^ College of Architecture and Material Engineering, Institute of Materials Research and Engineering (IMRE), Institute for the Application of Green Energy Materials, Hubei University of Education, Wuhan, China; ^2^ Key Laboratory of Catalysis and Energy Materials Chemistry of Ministry of Education, Hubei Laboratory of Catalysis and Materials Science, College of Chemistry and Materials Science, South-Central University of Nationalities, Wuhan, China; ^3^ College of Automotive Technology and Service, Wuhan City Polytechnic, Wuhan, China; ^4^ Key Laboratory for Green Chemical Process of Ministry of Education, School of Chemical Engineering and Pharmacy, Wuhan Institute of Technology, Wuhan, China

**Keywords:** biomass conversion, photocatalytic oxidation, metalloporphyrazine, glucose, glucaric acid

## Abstract

Photocatalysis has exhibited huge potential in selective conversion of glucose into value-added chemicals. Therefore, modulation of photocatalytic material for selective upgrading of glucose is significant. Here, we have investigated the insertion of different central metal ions, Fe, Co, Mn, and Zn, into porphyrazine loading with SnO_2_ for access to more efficient transformation of glucose into value-added organic acids in aqueous solution at mild reaction conditions. The best selectivity for organic acids containing glucaric acid, gluconic acid, and formic acid of 85.9% at 41.2% glucose conversion was attained by using the SnO_2_/CoPz composite after reacting for 3 h. The effects of central metal ions on surficial potential and related possible factors have been studied. Experimental results showed that the introduction of metalloporphyrazine with different central metal ions on the surface of SnO_2_ has a significant effect on the separation of photogenerated charges, changing the adsorption and desorption of glucose and products on the catalyst surface. The central metal ions of cobalt and iron contributed more to the positive effects toward enhancing conversion of glucose and yields of products, and manganese and zinc contributed more to the negative effects, resulting in the poor yield of products. The differences from the central metals may attribute to the surficial potential change of the composite and the coordination effects between the metal and oxygen atom. An appropriate surficial potential environment of the photocatalyst may achieve a better interactive relationship between the catalyst and reactant, while appropriate ability of producing active species matched with adsorption and desorption abilities would gain a better yield of products. These results have provided valued ideas for designing more efficient photocatalysts in selective oxidation of glucose utilizing clean solar energy in the future.

## 1 Introduction

Biomass valorization into value-added chemicals and fuels has been widely acknowledged as a sustainable approach in alleviating environmental issues and energy crises (Zhang et al., 2016; Hu et al., 2020; Wu et al., 2020; Wu et al., 2021; Yuan et al., 2021). Glucose, one of the most plentiful biomass-derived monosaccharides, has been transformed into a series of value-added products, like gluconic acid, glucaric acid, and formic acid (Choudhary et al., 2013; Colmenares et al., 2013; Liu et al., 2020). Gluconic acid is an important commercial chemical, which is widely used in many fields, such as food additives, medical intermediates, and the precursor of polymer synthesis (Ramachandran et al., 2006; Corma et al., 2007; Deng et al., 2014). Glucaric acid, ranked as one of the “top value-added chemicals from biomass” by the US Department of Energy, has also been introduced into a wide range of applications (Bozell and Petersen, 2010). It is a key building block for the production of various functional polymers, including new nylons and hyperbranched polyesters from biomass, and a potential alternative for polyphosphates in detergents. Its derivatives have also been studied for therapeutic purposes, such as the hormone-regulating agent for cancer prevention (Bozell and Petersen, 2010; Jin et al., 2016; Lee et al., 2016). In addition, formic acid has also been valued as an excellent hydrogen carrier in the context of a hydrogen energy economic picture because formic acid dehydrogenation can easily proceed under mild conditions using catalysts (Fukuzumi et al., 2010; Tedsree et al., 2011; Guerriero et al., 2014). The traditional process of gluconic acid and glucaric acid production involved bio-catalysis and chemo-catalysis approaches, wherein the process has not only consumed high energy but also faced the harm of noble metal poisoning (Smith et al., 2012; Derrien et al., 2017; Solmi et al., 2017; Xu et al., 2017; Chen et al., 2018; Zhang et al., 2020). Alternatively, photocatalysis has received growing attention in recent years, especially in energy and environmental applications (Zhang et al., 2019; Ma et al., 2020; Li et al., 2021; Ma et al., 2021). Although with great promises, selective conversion of biomass into value-added bioproducts *via* traditional photocatalysts is still challenging.

Having been well known for their extensive absorption in the visible light region and excellent electron transfer performance in natural redox systems, metalloporphyrins show their potential in optical utilization for photocatalysis. Among the metalloporphyrins, metalloporphyrazines bearing sulfur-containing groups, abbreviated to metallothioporphyrazines, in the macrocyclic periphery exhibit unique electronic and optical properties in comparison with their porphyrin counterparts. Therefore, sulfur-containing porphyrazine [Pz(Sbu)_8_] has shown better photocatalytic efficiency and has even found to be active in a nitrogen atmosphere in the degradation of organic pollutants (Jin et al., 2018; Li et al., 2018). In the previous work, we have reported that the strong interaction between metalloporphyrazine and SnO_2_ promoted conversion and selectivity of photocatalytic glucose oxidation under simulated sunlight irradiation (Zhang et al., 2019). Porphyrazine with sulfur-containing peripheral groups and the central metal ion iron has efficiently changed the photocatalytic behavior of SnO_2_ and improved selectivity toward organic acids. Thus, Pz(Sbu)_8_ has been verified as a possible photocatalyst for promoting biomass transformation more efficiently. In the follow-up studies (Zhang et al., 2020; Zhang et al., 2021), surficial modification of porphyrazine loading on the catalyst has been found to change the potential of the photocatalyst and made sense in improving the interactive relationship among glucose, products, and catalysts, which may be an important factor for selectivity enhancement. Therefore, appropriate modification for catalysts based on porphyrazine, like central metal ions, may also be a good attempt for selective oxidation of glucose. To the best of our knowledge, no similar research on modulation of central metal ions in metalloporphyrazines for glucose conversion has been investigated.

Herein, modification research of porphyrazine-based photocatalysts has been carried out *via* changing the central metal ion of metalloporphyrazine loaded on SnO_2_ for selective oxidation of glucose. The selectivity and yields of gluconic acid and glucaric acid are promoted. Through comparison of glucose conversion and selectivity of organic acids among the central metal ions of iron, cobalt, zinc, and manganese, the optimal central metal of porphyrazine has been selected, with which the total yield of value-added organic acids of the glucose could reach nearly 40% at ambient conditions. Meanwhile, the effects of glucose concentration, light intensity, and related possible factors have been studied. Its mechanism of photocatalytic oxidation was also explored. Results of absorption experiments and zeta potential characterization indicated that central metal modulation of the composite photocatalyst showed positive effects on enhancing the selectivity of gluconic acid and glucaric acid, which may break away more quickly from a more negative potential central metal oxidation. Originated from central metal modulation, surficial potential changing of the photocatalyst may be a potential approach for efficient and green conversion of glucose to high value-added chemicals.

## 2 Experimental section

### 2.1 Materials and instruments

In this work, all chemicals and solvents used were procured from Aladdin Chemicals Co., Ltd. and Sinopharm Chemical Reagent Co., Ltd., and all of them were of analytical grade and used without further purification.

The X-ray diffraction (XRD) patterns were measured on a D8 ADVANCE X-ray diffractometer with Cu-Kα radiation. Ultraviolet-visible diffuse reflectance spectral (UV-vis DRS) measurements were determined using a Shimadzu UV-2600 UV-vis spectrophotometer, and BaSO_4_ was used as a reference material. X-ray photoelectron spectroscopy (XPS) measurements were carried out by using a VG MultiLab 2000 spectrometer, and all binding energies were calibrated by using the adventitious C1s peak at 284.8 eV as a reference. The high-angle annular dark-field scanning TEM (HAADF-STEM) image and the corresponding element mapping of sample were obtained from a FEI Talos F200X Scanning Transmission Electron Microscope (STEM). Raman spectra were achieved using the DXR2xi Raman Imaging Microscope with a laser of wavelength 536 nm . Electron spin resonance (ESR) spectra were collected with a JES-FA200 spectrometer.

### 2.2 Preparation of tetra (2,3-bis(butylthio)-maleonitrile)porphyrazine with iron, cobalt, zinc, and manganese (abbreviated to MPz, M = Fe^2+^, Co^2+^, Zn^2+^, and Mn^2+^)

The chemical structures of metalloporphyrazines are shown in Supplementary Figure S1. Metalloporphyrazines with different central metal ions were synthesized and characterized, as previously described (Zhou et al., 2017; Zhang et al., 2019). First, tetra (2,3-bis (butylthio)-maleonitrile) porphyrazine [H_2_Pz(Sbu)_8_] was prepared by the following procedure. 2,3-Bis(butylthio)maleonitrile (0.5 g) was added to magnesium butoxide (100 mL); then, the mixture was stirred under reflux for 48 h in a nitrogen atmosphere. After that, the crude product was obtained by filtration and then washed with methanol until the filtrate was colorless. In order to remove the central metal, the obtained product was added to trifluoroacetic acid (50 mL) and stirred for 12 h in the dark. After removing the trifluoroacetic acid, dark purple products were obtained, which were further purified by column chromatography with silica gel, the eluent was dichloromethane/petroleum ether (v/v, 3:1). The target product [H_2_Pz(Sbu)_8_] was characterized with the ^1^H NMR spectrum and MALDI-TOF MS. The characteristic structure dates for the target products are as follows: the yield was 60% (1.2 g); ^1^H NMR spectrum (in CDCl_3_): 4.10–4.14 (t, 16H), 1.89 (m, 16H), 1.67 (m, 16H), 0.90–0.97 (t, 24H), and −1.12 (s, 2H); and MALDI-TOF HRMS: m/z = 1019.547 [M + H]^+^ (Supplementary Figures S2, S3).

Subsequently, under a nitrogen atmosphere, H_2_Pz(Sbu)_8_ (0.1 mmol) was heated with related metallic acetate (0.5 mmol) in 50 mL DMF at 70 °C for 10 h. After removing most of the solvent, the mixture was poured into dichloromethane (20 mL). The precipitate was collected by filtration and chromatographed on silica gel using hexane/DCM (v/v, 3:1) as the eluent. The related product (MPz, M = Fe^2+^, Co^2+^, Zn^2+^, and Mn^2+^) was obtained and characterized with MALDI-TOF MS, UV-vis spectrum in Supplementary Figures S4–S7. FePz: the yield was 51.4% (55 mg). MALDI-TOF HRMS: m/z = 1074.743 [M + H]^+^. CoPz: the yield was 78.9% (85 mg). MALDI-TOF HRMS: m/z = 1078.223 [M + H]^+^. ZnPz: the yield was 64.6% (70 mg). MALDI-TOF HRMS: m/z = 1084.639 [M + H]^+^. MnPz: the yield was 21.4% (23 mg). MALDI-TOF HRMS: m/z = 1107.635 [M + H]^+^.

### 2.3 Preparation of SnO_2_/MPz (M = Fe^2+^, Co^2+^, Zn^2+^, and Mn^2+^)

In a typical fabrication procedure for preparing the SnO_2_/CoPz composite, 10 mg of CoPz was first dissolved into 50 mL of dichloromethane, obtaining a solution of CoPz in dichloromethane. Subsequently, 2 g of commercial SnO_2_ was added to the foregoing solution. After sonicating for 30 min, the mixture was stirred for 12 h at room temperature. The resulting SnO_2_/CoPz composite was obtained by removing the solvent with reduced pressure distillation. This sample was denoted as SnO_2_/CoPz. A range of SnO_2_/MPz composites with different porphyrazines of MPz, such as FePz, ZnPz, and MnPz, were also prepared using the same procedure as the SnO_2_/CoPz sample through the addition of different metalloporphyrazines during preparation, and these samples were correspondingly denoted as SnO_2_/FePz, SnO_2_/ZnPz, and SnO_2_/MnPz, respectively. The content of MPz in all SnO_2_/MPz composites was about 0.5%.

### 2.4 Photocatalytic oxidation of glucose

Typically, 20 mg of the photocatalyst was suspended in 30 mL of prepared aqueous glucose at a certain concentration in a cylindrical vessel with a circling water-cooled jacket. Subsequently, the reaction solution was continuously stirred for 30 min in the dark to ensure the establishment of an adsorption–desorption equilibrium between the photocatalyst and glucose. The reaction solution was magnetically stirred before and during the illumination. Atmospheric dioxygen was used directly as an oxidant in the photocatalytic reaction, so the vessel was directly opened to the atmosphere under ambient conditions. Water circulated in the circling water-cooled jacket, which controlled the photocatalytic reaction at room temperature. A xenon lamp (Beijing China Education Au-light Co., Ltd.) was used as the irradiation source for providing simulated sunlight irradiation, and the light intensity was 1.5 W·cm^−2^ unless otherwise specified. As the photocatalytic reaction proceeded, 1 mL of the suspension was drawn from the system at a certain time interval and filtered through a filter membrane to remove the photocatalyst.

According to Jin et al. (2013), the obtained filtrate was analyzed using a Dionex UltiMate 3000 HPLC system with a refractive index (ERC RefractoMax 520) detector to determine the concentration of each product. Analyses were conducted using 0.4 mmol·L^−1^ H_2_SO_4_ solution as the mobile phase with a flow rate of 0.6 mL·min^−1^. The injection volume of the sample was 20 μL. The Shodex SUGAR SH1011 column was used for the analytical separation of products, and the column was thermostated at 50 °C for the separation. Product identification was made by the comparison of HPLC retention times with authentic samples and by LC–MS analysis; a Shimadzu LCMS-8050 triple quadrupole mass spectrometer was used for the LC–MS analysis of samples. The conversion and selectivity for photocatalytic oxidation of glucose were defined as the following:

$$Conversion=\frac{C_{0}{-C}_{r}}{C_{0}}\times 100\%,$$

$$Selectivity=\frac{C_{p}}{C_{0}{-C}_{r}}\times 100\%,$$

where C_*0*_ is the original concentration of glucose, and C_*r*_ and C_*p*_ are the concentrations of the detected glucose and the corresponding oxidation product at a certain time after the photocatalytic reaction, respectively.

## 3 Results and discussion

### 3.1 Catalyst characterization

**FIGURE 1:**
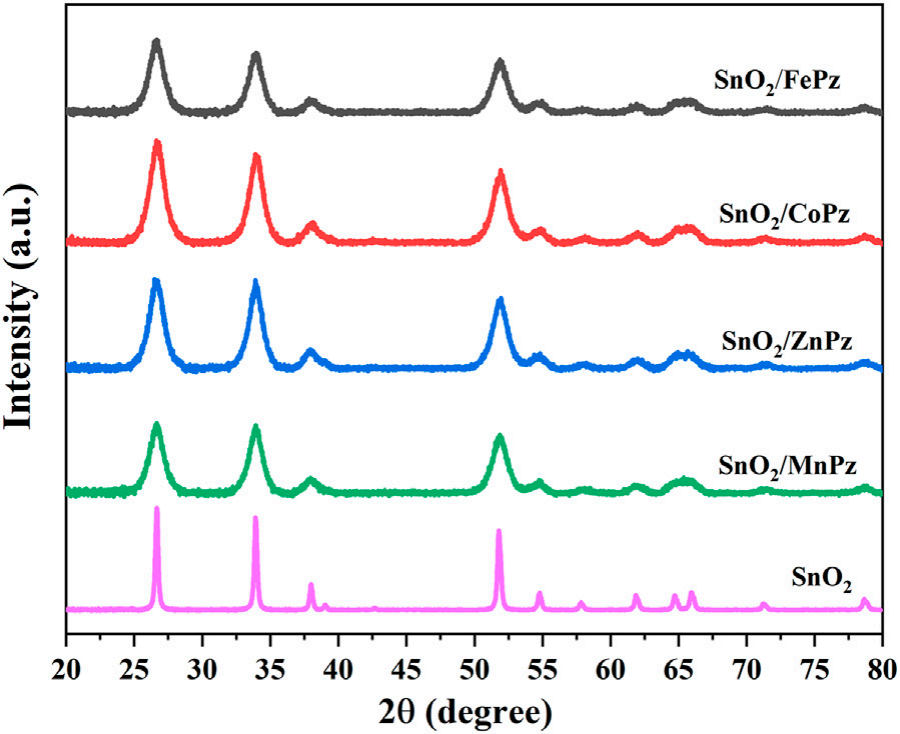
X-ray diffraction (XRD) patterns of SnO_2_/MPz composites (M = Fe^2+^, Co^2+^, Zn^2+^, and Mn^2+^).

**FIGURE 2:**
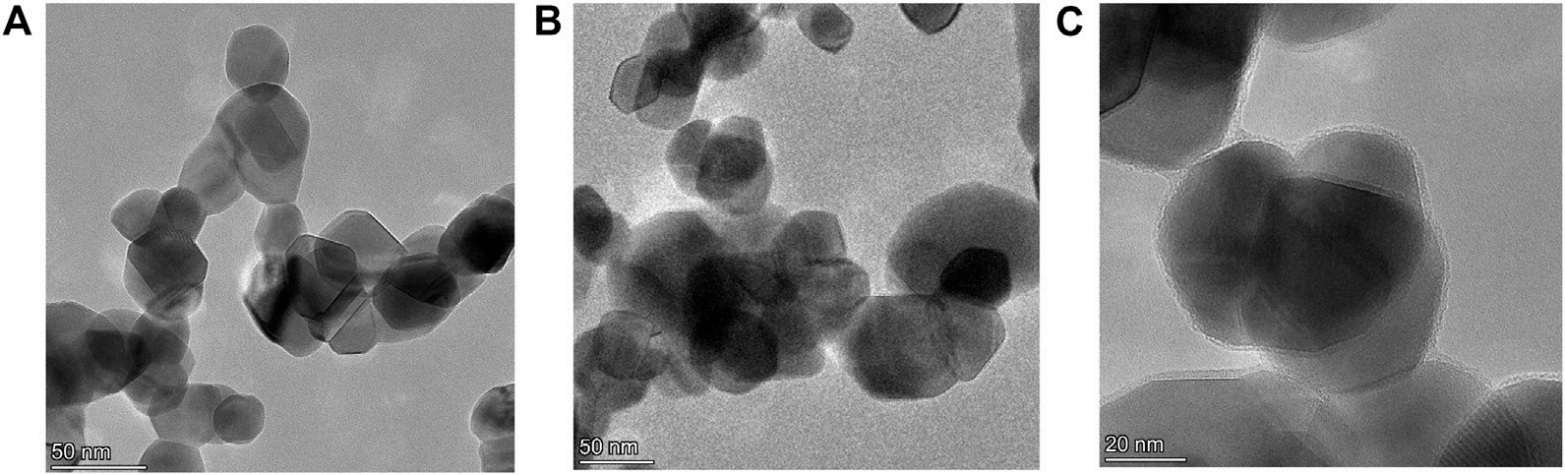
HAADF-STEM images of SnO_2_ **(A)** and the SnO_2_/MPz composite **(B, C)**.

XRD patterns were analyzed to provide information on the crystallographic structure of photocatalysts. Figure 1 shows the XRD patterns of SnO_2_ and SnO_2_/MPz composites with different central metals. The as-prepared SnO_2_ presented the characteristic diffraction peaks at 2*θ* of 26.7°, 33.8°, 38.0°, 51.9°, 61.9°, and 65.7°, assigned to the (110), (101), (200), (211), (310), and (112) crystallographic planes of rutile type SnO_2_ (Yang et al., 2018). A mildly broadened diffraction peak for SnO_2_ manifests that SnO_2_ nanoparticles are small. The XRD pattern of SnO_2_/MPz composites was similar to that of SnO_2_. These results illustrate that the introduction of metalloporphyrazines has no influence on the bulk intrinsic structure of SnO_2_. Moreover, the STEM images of SnO_2_ and SnO_2_/MPz are shown in Figure 2. The morphology of SnO_2_ remained essentially unchanged before and after loading with metalloporphyrazines, which is in good agreement with the XRD analysis. However, a film-like layer was clearly presented on the surface of SnO_2_ in the SnO_2_/MPz composite in Figure 2C, which should be the deposited metalloporphyrazines. Therefore, it can be confirmed that metalloporphyrazine molecules were successfully impregnated onto the surface of SnO_2_.

**FIGURE 3:**
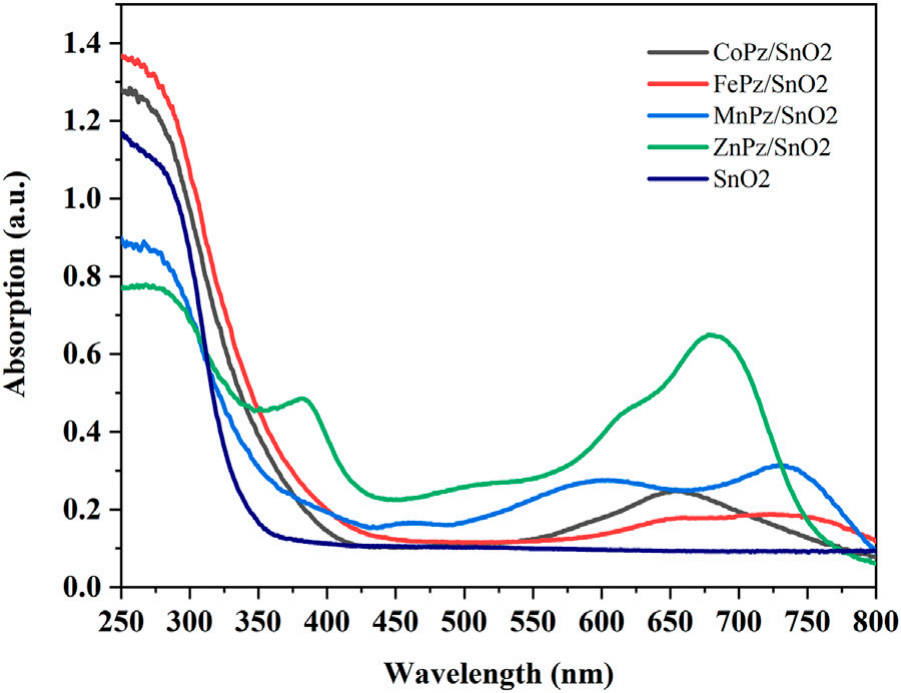
UV-vis diffuse reflectance spectra (DRS) of SnO_2_/MPz composites.

The optical property of photocatalyst is of great importance to its photocatalytic performance. The light absorption properties of pure SnO_2_ and SnO_2_/MPz composites with different central metals were monitored by UV-vis diffuse reflectance spectra (DRS), as shown in Figure 3. Pure SnO_2_ displays weak absorption in the range of a visible light wavelength above 400 nm. It was noteworthy that the SnO_2_/CoPz composite exhibited a strong visible light absorption located at about 653 nm, which could be ascribed to the Q-band of CoPz. The Q-band of porphyrazine corresponds to the π→π* transition of the conjugated π–electron macrocycle. In comparison with the central metal ion of cobalt, other metals both have showed similar photo absorption ability in both UV light and visible light regions.

**FIGURE 4:**
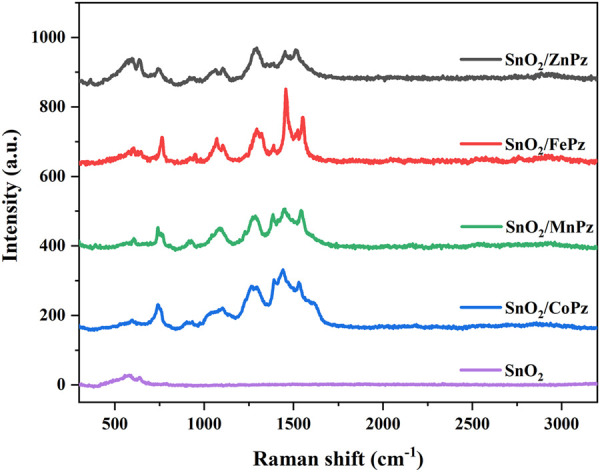
Raman spectra of SnO_2_ and SnO_2_/MPz (M = Fe^2+^, Co^2+^, Zn^2+^, and Mn^2+^) composites.

Raman spectroscopy, as one of the versatile tools, was further used to identify the modification of porphyrazine induced at the surface of SnO_2_. The Raman spectra of SnO_2_ and SnO_2_/MPz composites are presented in Figure 4. From the Raman spectrum of SnO_2_ in Figure 4, a wide peak appears in the 400–800 cm^−1^ region, which can be resolved into two peaks at about 625 and 575 cm^−1^. The peak at 625 nm corresponded to the symmetric A1 g stretching vibration mode of the Sn-O bond of SnO_2_. In the CoPz/SnO_2_ curve, the characteristic peaks of Co-N (598 cm^−1^), C-H (756 cm^−1^), C-S (939 cm^−1^), C-C (1055 and 1097 cm^−1^), and C=C (1284, 1309, 1448, 1508, and 1541 cm^−1^) vibrations originated from the introduction of CoPz. There were no significant differences in other central metal curves for similar structures. All the characteristic bands of metalloporphyrazines presented in the SnO_2_/MPz composite. In addition, compared with the characteristic peaks of pure CoPz, the corresponding peaks of CoPz in the SnO_2_/CoPz composite appeared as a blue shift, as shown in Supplementary Figure S8, indicating that there existed an interaction between SnO_2_ and CoPz. In comparison with other central metals, there is no significant shift besides the characteristic peaks of metalloporphyrazines.

**FIGURE 5:**
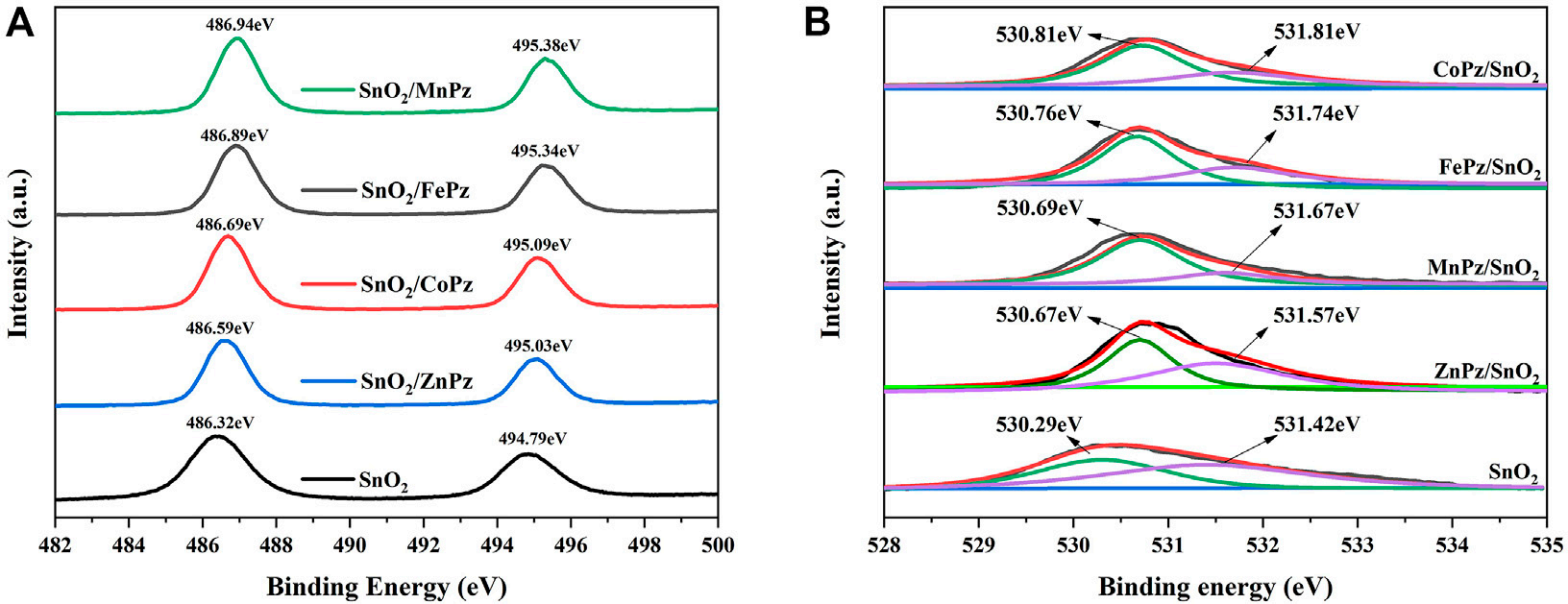
High-resolution XPS spectra of SnO_2_ and SnO_2_/MPz (M = Fe^2+^, Co^2+^, Zn^2+^, and Mn^2+^) composites. **(A)** the high-resolution XPS of spectra of Sn 3d, **(B)** the high-resolution XPS of spectra of O 1s.

Subsequently, X-ray photoelectron spectroscopy (XPS) was performed to further study the chemical environment changes of Sn and O with SnO_2_ before and after supporting metalloporphyrazines. As shown in Figure 5A, two peaks at a binding energy of 486.32 and 494.79 eV are observed for SnO_2_, which correspond to Sn 3d_5/2_ and Sn 3d_3/2_, respectively. Figure 5B shows the high-resolution XPS of spectra of O 1s, SnO_2_ displays two oxygen peaks, 530.29 and 531.42 eV, which correspond to the O 1s region, namely, the former can be ascribed to O^2−^ of lattice oxygen and the latter can be ascribed to chemisorbed oxygen (Vià et al., 2017). When metalloporphyrazines were introduced onto the surface of SnO_2_, both the peaks of Sn 3d and O 1s were shifted to a higher energy. The shifts indicated a robust interfacial contact between SnO_2_ and metalloporphyrazines (Zhang et al., 2019). Furthermore, the binding energies of Sn 3d and O 1s have shown correlation with the central metal. Among these central metals, lattice oxygen and chemisorbed oxygen bound to cobalt and iron have shown higher binding energies than other metal coordination. Combining with the results obtained from Raman spectra, the tight combination of SnO_2_ with CoPz and FePz can validly facilitate the transfer of electron and energy during the photocatalytic process.

### 3.2 Photocatalytic oxidation of glucose

As shown in Table 1, photocatalytic oxidation of glucose with photocatalysts has been carried out under simulated sunlight irradiation on using atmospheric dioxygen as an oxidant in aqueous solution. No glucose conversion was observed without light irradiation or the photocatalyst (Table 1, Entry 1). Under light irradiation, SnO_2_ alone gives 11.3% conversion of glucose in an aqueous medium; the value-added organic acids including glucaric acid, gluconic acid, and formic acid are obtained with a total selectivity of 47.2% (Table 1, Entry 3). As previous results, after introducing the metalloporphyrazines onto the surface of SnO_2_, glucose conversion was obtained with apparent promotion. With loading MnPz onto SnO_2_, the glucose conversion slightly went up to 16.9%, while value-added products got less than pure SnO_2_ with a total selectivity of 18.9%. In comparison with MnPz, ZnPz loading with SnO_2_ improved the conversion of glucose, 27.3%, maintaining similar selectivity with pure SnO_2_. The highest glucose conversion of 41.2% was obtained with CoPz loading onto SnO_2_, while the total selectivity of organic acids reached up to 85.9% (Table 1, Entry 4). It was indicated that the introduction of CoPz was not only beneficial to the conversion of glucose but also to the formation of organic acids, further making clear that the presence of CoPz can play a positive role on the photocatalytic oxidation of glucose. As for the central metal ion of iron in porphyrazine on SnO_2_, the glucose conversion and selectivity have showed to be apparently increased than pure SnO_2_, second only to CoPz (Table 1, Entry 5). A comparison between central metal ions of cobalt and iron was performed; the selectivity of both showed nearly 80%, while the conversion of SnO_2_/CoPz gained slightly higher 7% than SnO_2_/FePz. It is clear that the central metals of porphyrazine have different effects on the performance of composite photocatalysts in selective oxidation of glucose. Based on the previous analysis, the synergistic action between metalloporphyrazine and SnO_2_ has been speculated to vary with central metals changing for photocatalytic oxidation of glucose to organic acids.

**TABLE 1:** Photocatalytic oxidation of glucose under different conditions^a^.

Entry	Catalyst	Conversion (%)	Selectivity (%)			
			GAA	GOA	FA	TOA
1	Without	—	—	—	—	—
2^b^	SnO_2_	—	—	—	—	—
3	SnO_2_	11.3	11.2	21.1	14.9	47.2
4	SnO_2_/CoPz	41.2	19.8	51.2	5.9	85.9
5	SnO_2_/FePz	34.1	24.8	49.8	2.9	77.5
6	SnO_2_/ZnPz	27.3	12.6	27.9	2.8	43.3
7	SnO_2_/MnPz	16.9	5.2	11.2	2.5	18.9
8	TiO_2_(P25)^(Zhang et al., 2019)^	38.3	—	4.1	16.8	20.9
9	ZnO^(Zhang et al., 2019)^	5.9	—	10.6	—	10.6

^a^ Reaction conditions: aqueous glucose (1 mmol·l^−1^, 30 mL), catalyst (20 mg), reaction for 3 h, and light intensity (1.5 W·cm^−2^). (Note: GAA, glucaric acid; GOA, gluconic acid; FA, formic acid).

^b^ Without light irradiation. (Note: GAA, glucaric acid; GOA, gluconic acid; FA, formic acid; TOA, total organic acid).

**FIGURE 6:**
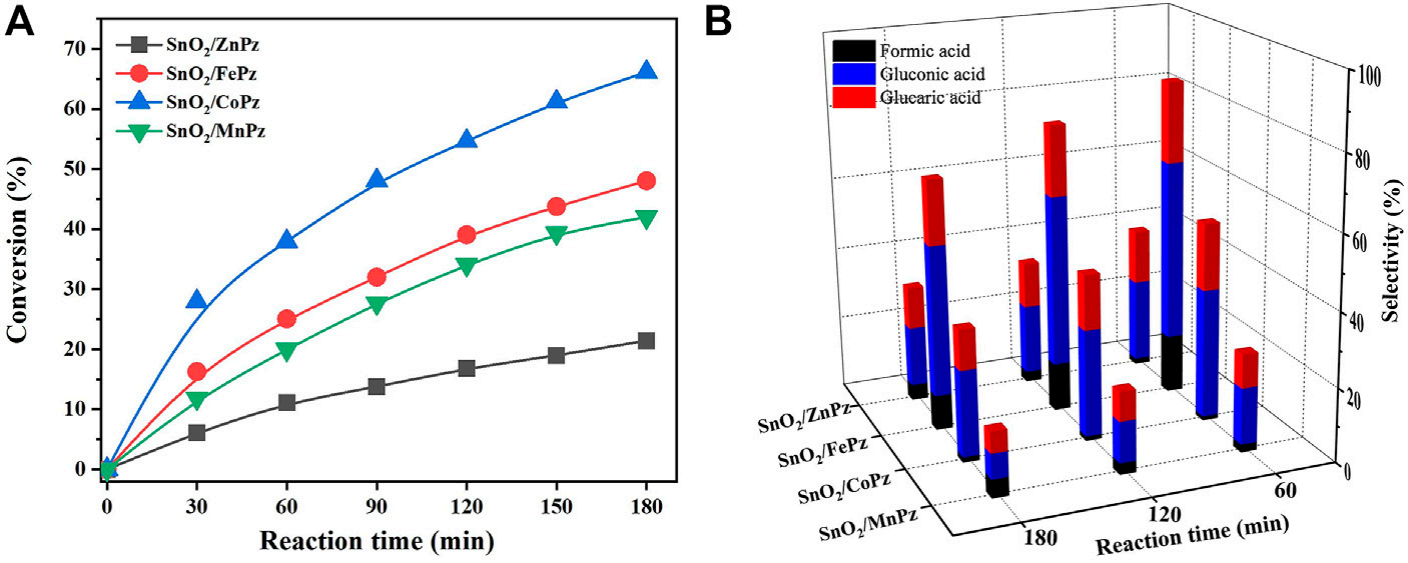
Conversion of glucose **(A)** and the selectivity of organic acid **(B)** over the SnO_2_/MPz composite with different central metal ions. Reaction conditions: aqueous glucose (0.5 mmol·L^−1^, 30 mL), catalyst (20 mg), and light intensity (1.5 W·cm^−2^).

**FIGURE 7:**
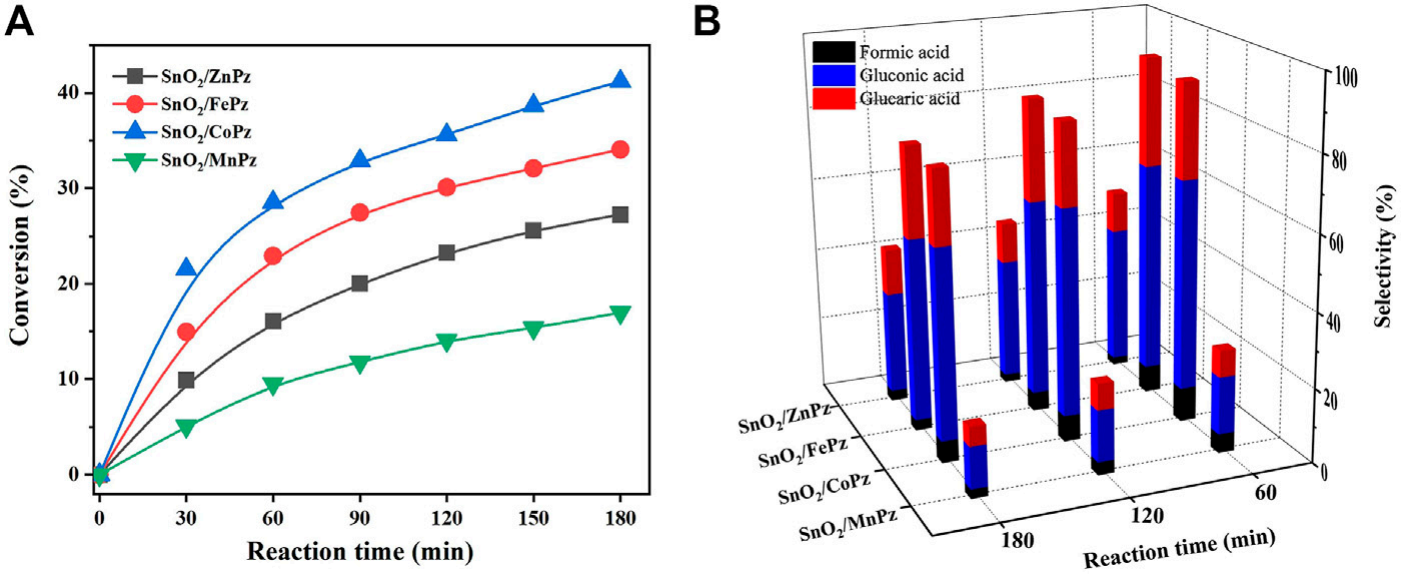
Conversion of glucose **(A)** and the selectivity of organic acid **(B)** over the SnO_2_/MPz composite with different central metals. Reaction conditions: aqueous glucose (1 mmol·L^−1^, 30 mL), catalyst (20 mg), and light intensity (1.5 W·cm^−2^).

**FIGURE 8:**
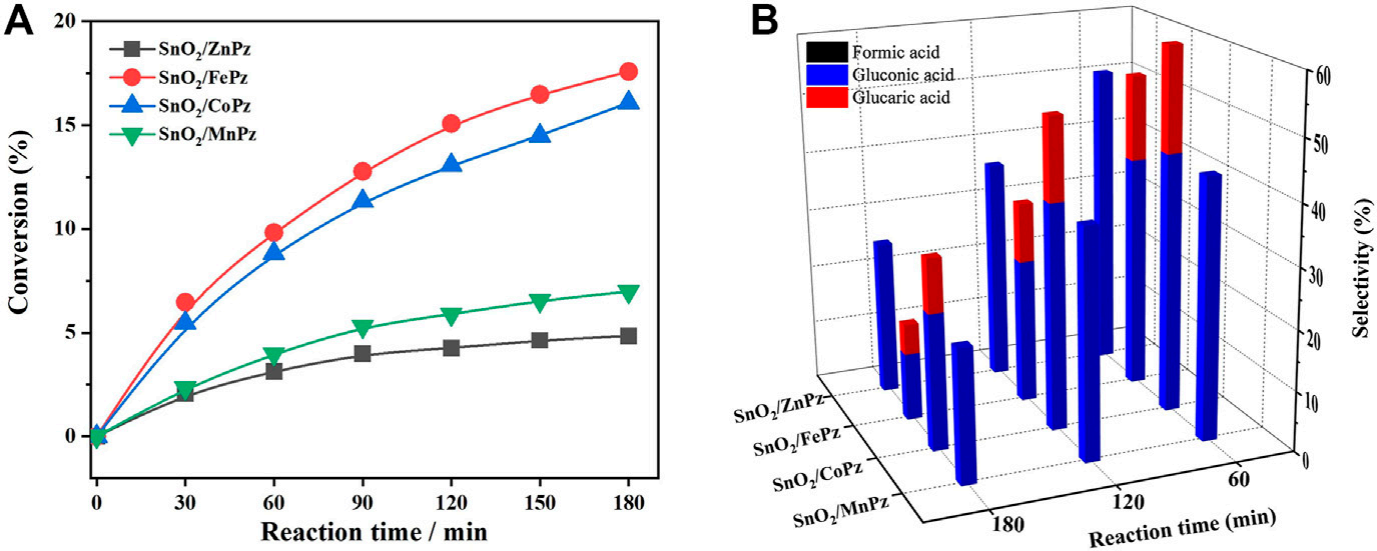
Conversion of glucose **(A)** and the selectivity of organic acid **(B)** over the SnO_2_/MPz composite with different central metal ions. Reaction conditions: aqueous glucose (4 mmol·L^−1^, 30 mL), catalyst (20 mg), and light intensity (1.5 W·cm^−2^).

In order to further illustrate the effect of central metal ions, the SnO_2_/MPz composite has been applied for photocatalytic selective oxidation of glucose under different glucose concentrations. As shown in Figure 6A, it can be seen that the glucose conversion increased gradually from zinc to cobalt in 0.5 mol·L^−1^ glucose solution. The highest conversion of glucose has reached 66.2% by the SnO_2_/CoPz composite, while total selectivity of organic acids has been 34.2%. In comparison with central metal ion of cobalt, SnO_2_/FePz has showed better performance in selectivity of organic acids, which has been achieved from 65% in conversion of 48.1%. The composite with zinc or manganese has obtained poor selectivity below 20%. When the concentration of glucose has been increased to 1 mol·L^−1^, as shown in Figure 7, glucose conversion of all SnO_2_/MPz composites both decreased. The performance of manganese has showed both poor conversion and selectivity below 20%. The glucose conversion ability of the zinc composite has not gained much, but the selectivity of organic acids has risen to 43.2%. The best conversion has been obtained in 41.2% *via* SnO_2_/CoPz, while the selectivity has also increased to 85.9% more than 77.5% in SnO_2_/FePz. According to the results, it is clear that the types of products have showed no relation with the central metal of porphyrazine in the SnO_2_/MPz composite, but the selectivity of organic acids has been affected strongly. In general, overoxidation of glucose would be intensified in a high conversion rate, which may lead in the decrease of selectivity for products. Based on the previous analysis, the decrease of selectivity may be related both with the conversion rate and the surficial relationship between the catalyst and glucose. When the concentration of glucose has been increased to 4 mol·L^−1^, as shown in Figure 8, the types of products with zinc and manganese have changed with no glucaric acid in a less conversion of glucose. It is speculated that less active species generated by zinc and manganese composites resulted in a low conversion rate, and no extra active species has been applied for deep oxidation of gluconic acid to glucaric acid. Therefore, the total selectivity has increased without overoxidation. In comparison between the central metal ions of cobalt and iron, SnO_2_/CoPz has showed better selectivity in gluconic acid and glucaric acid than SnO_2_/FePz, while the conversion of cobalt and iron has been similar. It is showed that the iron composite showed advantages of selectivity in low glucose concentrations, while the cobalt composite achieved better performance in a high glucose concentration. However, in 1 mol·L^−1^ glucose solution, the cobalt composite has got higher conversion and selectivity both at the same time. Furthermore, the effects of air atmosphere on SnO_2_/MPz composite photocatalysts have also been investigated. Compared with the results obtained from nitrogen conditions in Supplementary Figure S9, a much higher conversion of glucose and selectivity of total organic acids were observed in the presence of the SnO_2_/FePz composite photocatalyst than SnO_2_/CoPz, while no products have been detected through the central metals of zinc and manganese. Gluconic acid has been obtained both in SnO_2_ with CoPz and FePz. It is noted that glucaric acid has been obtained *via* FePz without enough oxygen for continuous activation. This indicated that the oxidation of the active intermediate in thioporphyrazines, like oxygen axially coordinated to central metal, could be applied for glucose conversion in all the metalloporphyrazines, but the active way in metalloporphyrazines with iron and cobalt for photocatalytic oxidation without oxygen would be distinct.

### 3.3 Mechanism of enhanced photoactivity

**FIGURE 9:**
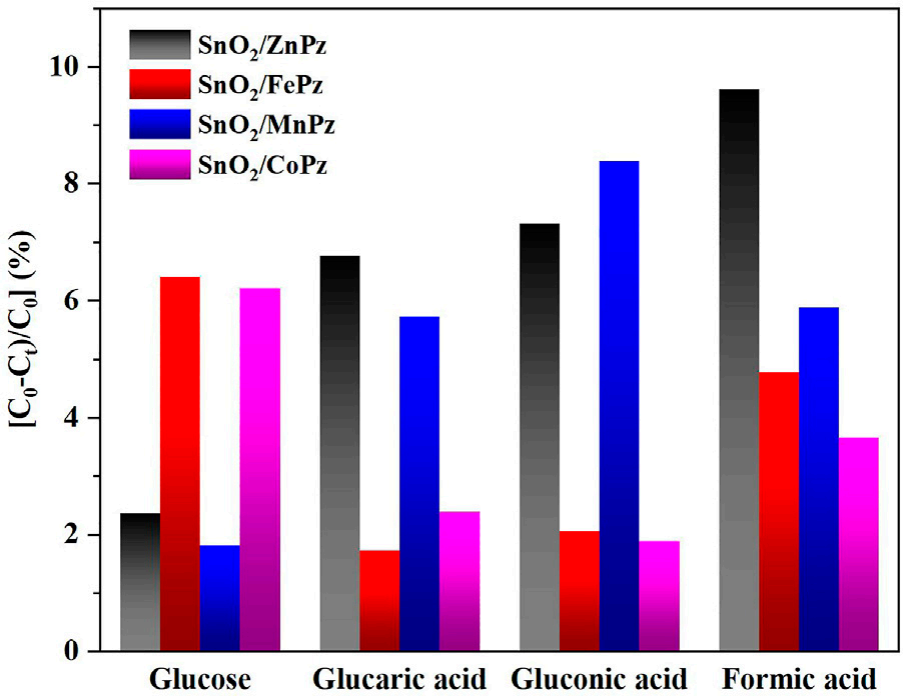
Adsorption of different substrates on the SnO_2_/MPz composite. The photocatalyst (20 mg) was immersed into 30 mL of solution containing glucose (0.06 mmol), glucaric acid (0.06 mmol), gluconic acid (0.06 mmol), and formic acid (0.06 mmol). The suspension system was stirred for 24 h in the dark at ambient temperature, and then, the filtered solution was used to analyze.

**FIGURE 10:**
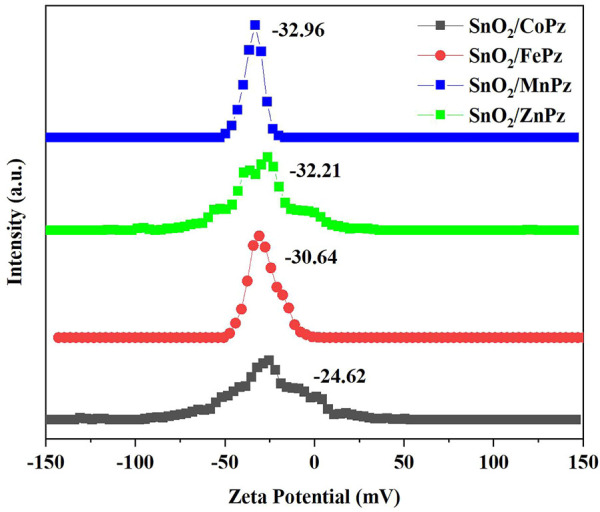
Zeta potential of SnO_2_ and SnO_2_/MPz composites with different central metal ions.

**FIGURE 11:**
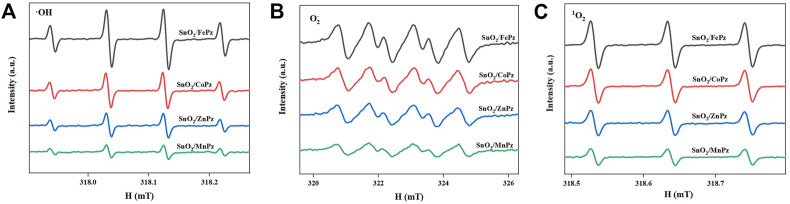
DMPO spin-trapping ESR spectra to detect •OH in aqueous solution **(A)** and to detect O_2_^•−^ in methanol solution **(B)**. TEMP spin-trapping ESR spectra to detect ^1^O_2_ in aqueous solution **(C)**. The ESR spin-trap measurements were performed in the presence of the SnO_2_/MPz composite under light irradiation for 10 min.

The aforementioned experimental results demonstrated that modulation of central metal ions in metalloporphyrazines in the SnO_2_/MPz composite led to a significant contribution to the performance in the photocatalytic oxidation of glucose to organic acids. Therefore, the related experiments were further implemented to elaborate on central metal ion effects in metalloporphyrazine-catalyzed selective oxidation of glucose. The adsorption of reactants and products on catalysts plays an important role in its catalytic activity. The adsorption experiments of glucose, glucaric acid, gluconic acid, and formic acid were conducted for different central metal composites, as shown in Figure 9. Pure SnO_2_ surrounding with porphyrazine of cobalt and iron has shown higher adsorption capacity of glucose than porphyrazine of zinc and manganese, indicating that the introduction of cobalt and iron can improve the adsorption of glucose on the surface of the photocatalyst. Moreover, it was observed that the adsorption of gluconic acid and glucaric acid over the porphyrazine composite of cobalt and iron apparently decreased, demonstrating that the surface has been changed by porphyrazine of cobalt and iron more repulsive toward glucaric acid and gluconic acid. The high adsorption capacity of glucose is beneficial to the mass transfer procedure, thus promoting the conversion of glucose. Among these different central metal composites, the difference in the adsorption capacity was greatest for glucaric acid and gluconic acid, while the difference in the adsorption capacity was minimal for formic acid. These results manifested that there exists a strong correlativity between the central metal ion and selectivity of organic acids. The previous results showed that the selectivity of gluconic acid over SnO_2_/CoPz (Table 1, Entry 4) was much higher than that in other metals, while the selectivity of glucaric acid over SnO_2_/FePz (Table 1, Entry 5) was much higher than that in other metals. The better performance maybe related to their weak adsorption and easy desorption *via* modification of the composite surface. For deeper research, the surface potential of composite photocatalysts with different central metal ions has been analyzed *via* zeta potential, which shows that the surface-absorbing ability related to electrostatic absorbing function. As expected, in Figure 10, the surface potential of the composite catalyst has shown apparent change when the central metal ion of porphyrazine has been switched. The gap reached its previous peak, nearly 8 mV, in porphyrazines of cobalt and manganese. The insertion of iron in the porphyrazine-based photocatalyst has modulated the surface electronegative values to −30.64 mV but less than porphyrazine of zinc and manganese, of which two of them have shown almost the same in surface potential of −32 mV. Combined with absorption results, it is indicated that appropriate electronegative values could increase the conversion of glucose and the selectivity of glucaric acid and gluconic acid. Moreover, the active species for photocatalytic oxidation of glucose among porphyrazine of different central metal ions was investigated by electron spin resonance (ESR), as shown in Figure 11. First, the obvious ESR signals of ·OH, O_2_·-, and ^1^O_2_ can be observed under light irradiation in both SnO_2_/MPz composites. It is indicated that all the composites produce the same main active species in the presence of atmospheric dioxygen in aqueous medium under light irradiation. However, the highest signals from SnO_2_/MnPz have results in poor selectivity, which may be because the strong ability of producing active species and the low desorption ability of products have enhanced overoxidation of products. The similar phenomenon has happened to a zinc composite. The appropriate ability of porphyrazine with iron and cobalt has obtained more gluconic and glucaric acids, indicating that higher active species yields have not been related with higher yields of value-added products. In other words, it can be concluded that the introduction of metalloporphyrazine on the surface of SnO_2_ has a significant effect on the separation of photogenerated charges and changing the adsorption and desorption of glucose and products on the catalyst surface. However, whether this effect has advantages may be changed *via* the modification of central metal ions in porphyrazine. The central metal ions of cobalt and iron contributed more to the positive effects toward enhancing conversion of glucose and yields of products, and the central metal ions of manganese and zinc contributed more to the negative effects, resulting in the poor yield of products. The differences from the central metals may belong to the surficial potential change of the composite and the coordination effects between the metal and oxygen atom.

## 4 Conclusion

In summary, several photocatalysts SnO_2_/MPz (M = Fe^2+^, Co^2+^, Zn^2+^, and Mn^2+^) were successfully prepared and well characterized by various technologies. In selective oxidation of glucose, the ability of conversion and yields of gluconic and glucaric acids have showed to be related with the modification of central metals. This work demonstrated that the porphyrazine-based photocatalyst with the central metal ions of cobalt and iron showed better performance toward gluconic and glucaric acids than zinc and manganese. Porphyrazine with cobalt central metal may prefer gluconic acid, while iron may prefer glucaric acid. The total selectivity for organic acids containing glucaric acid, gluconic acid, and formic acid of 85.9% at 41.2% glucose conversion was attained by using the SnO_2_/CoPz composite after reacting for 3 h. The effect of adsorption capacities of this composite photocatalyst for reactants and products on the oxidation of glucose was investigated in this work. The studies on the role of various active radicals in glucose oxidation showed that ·OH, O_2_·-, and ^1^O_2_ were the active intermediates in the photocatalytic oxidation process. According to the previous analysis, the appropriate surficial potential environment of the photocatalyst may achieve a better interactive relationship between the catalyst and reactant, while appropriate ability of producing active species matched with adsorption and desorption abilities would gain a better yield of products.

## Data Availability

The original contributions presented in the study are included in the article/Supplementary Material; further inquiries can be directed to the corresponding authors.
