# Corrigendum: Improving yields by switching central metal ions in porphyrazine-catalyzed oxidation of glucose into value-added organic acids with SnO_2_ in aqueous solution

**DOI:** 10.3389/fchem.2023.1271707

**Published:** 2023-09-26

**Authors:** Quanquan Zhang, Xin Li, Xingwang Wang, Xin Huang, Yuncai Liu, Fengshou Wu, Bingguang Zhang, Kejian Deng

**Affiliations:** ^1^ College of Architecture and Material Engineering, Institute of Materials Research and Engineering (IMRE), Institute for the Application of Green Energy Materials, Hubei University of Education, Wuhan, China; ^2^ Key Laboratory of Catalysis and Energy Materials Chemistry of Ministry of Education, Hubei Laboratory of Catalysis and Materials Science, College of Chemistry and Materials Science, South-Central University of Nationalities, Wuhan, China; ^3^ College of Automotive Technology and Service, Wuhan City Polytechnic, Wuhan, China; ^4^ Key Laboratory for Green Chemical Process of Ministry of Education, School of Chemical Engineering and Pharmacy, Wuhan Institute of Technology, Wuhan, China

**Keywords:** biomass conversion, photocatalytic oxidation, metalloporphyrazine, glucose, glucaric acid

In the published article, there were errors in the **Author** list. The author “Xin Li” was erroneously excluded, and the order of authors was incorrect. The corrected author list appears above.

In the published article, some **Affiliations** were erroneously attributed to the incorrect authors. The correct **Affiliations** appear above.

The authors apologize for these errors and state that this does not change the scientific conclusions of the article in any way. The original article has been updated.

