# Supplementary material for: Improving yields by switching central metal ions in porphyrazine-catalyzed oxidation of glucose into value-added organic acids with SnO2 in aqueous solution
Source: Front Chem. 2023 May 30;11:1114454. doi: 10.3389/fchem.2023.1114454 (PMC10266338; doi:10.3389/fchem.2023.1114454)
Supplement: Supplementary file 1 [file DataSheet1.DOCX]

Supplementary Material

Supplementary Figure S1. Chemical structrues of tetra(2,3-bis(butylthio)-maleonitrile) porphyrazine (H_2_Pz(Sbu)_8_) and tetra(2,3-bis(butylthio)-maleonitrile)porphyrazine with Iron, Cobalt, Zinc, Manganese (MPz, M= Fe^2+^, Co^2+^, Zn^2+^ and Mn^2+^)


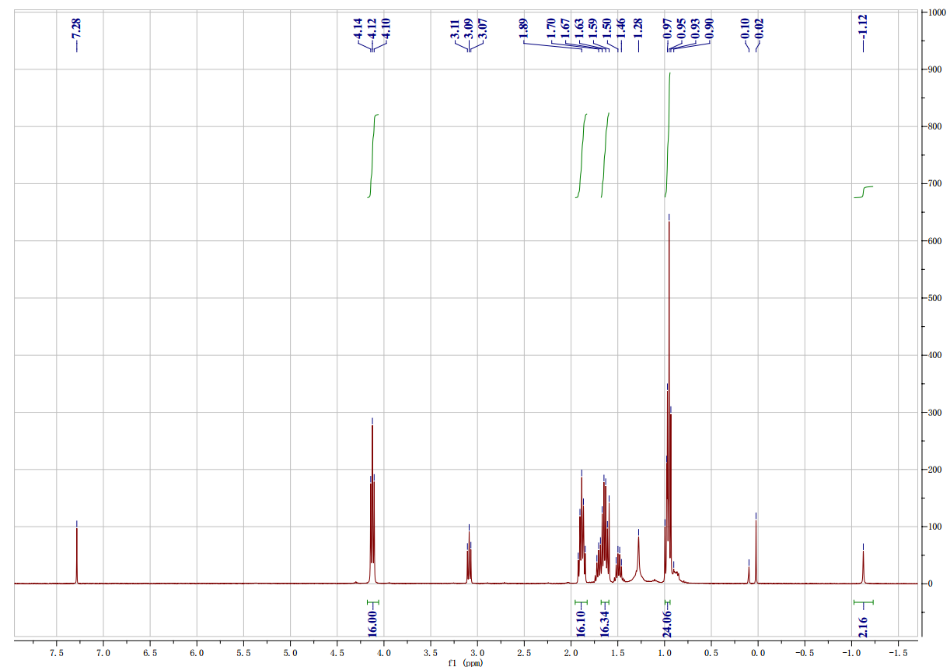


Supplementary Figure S2. ^1^H NMR spectra of tetra(2,3-bis(butylthio)-maleonitrile) porphyrazine (H_2_Pz(Sbu)_8_).


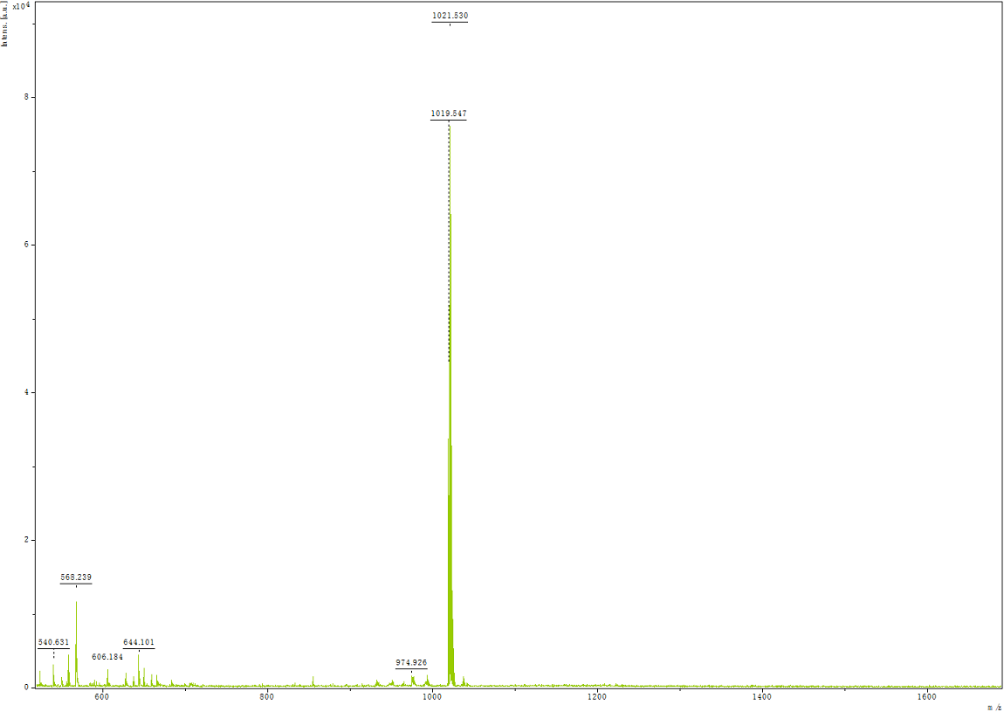


Supplementary Figure S3. TOF-MALDI-MS of tetra(2,3-bis(butylthio)-maleonitrile) porphyrazine (H_2_Pz(Sbu)_8_).


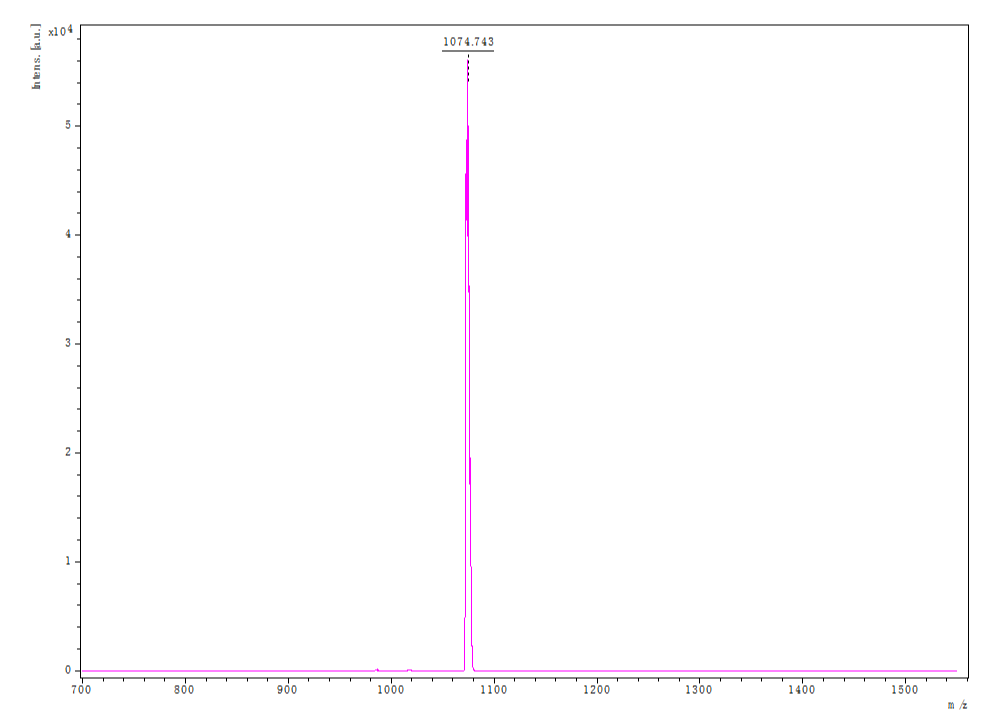


Supplementary Figure S4. TOF-MALDI-MS of tetra(2,3-bis(butylthio)-maleonitrile)porphyrazine with Iron (FePz(Sbu)_8_).


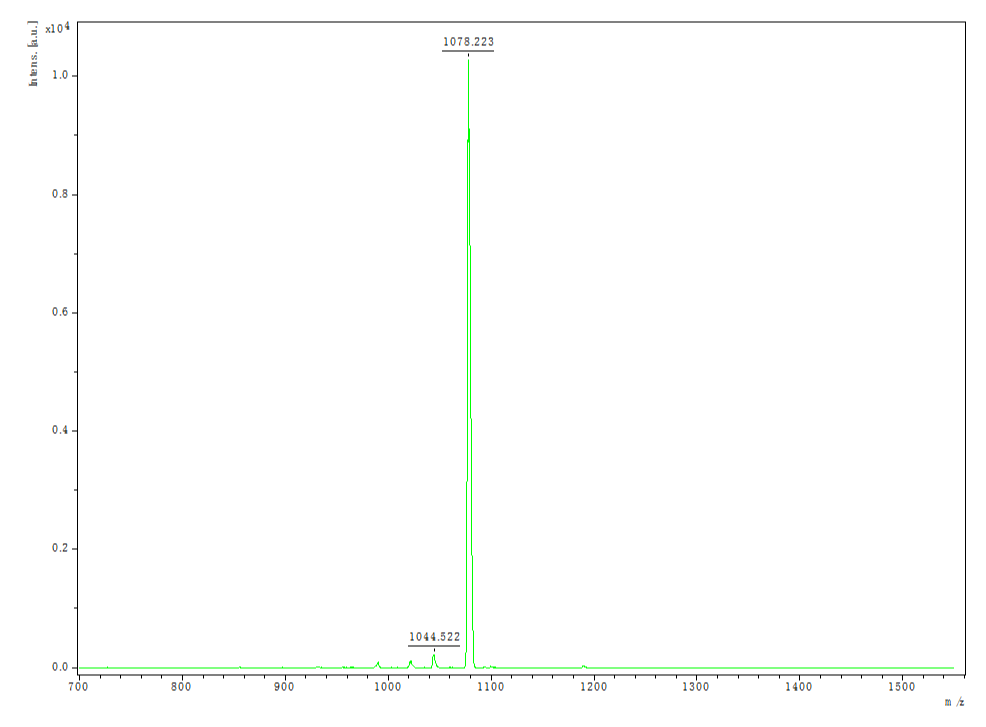


Supplementary Figure S5. TOF-MALDI-MS of tetra(2,3-bis(butylthio)-maleonitrile)porphyrazine with Cobalt (CoPz(Sbu)_8_).


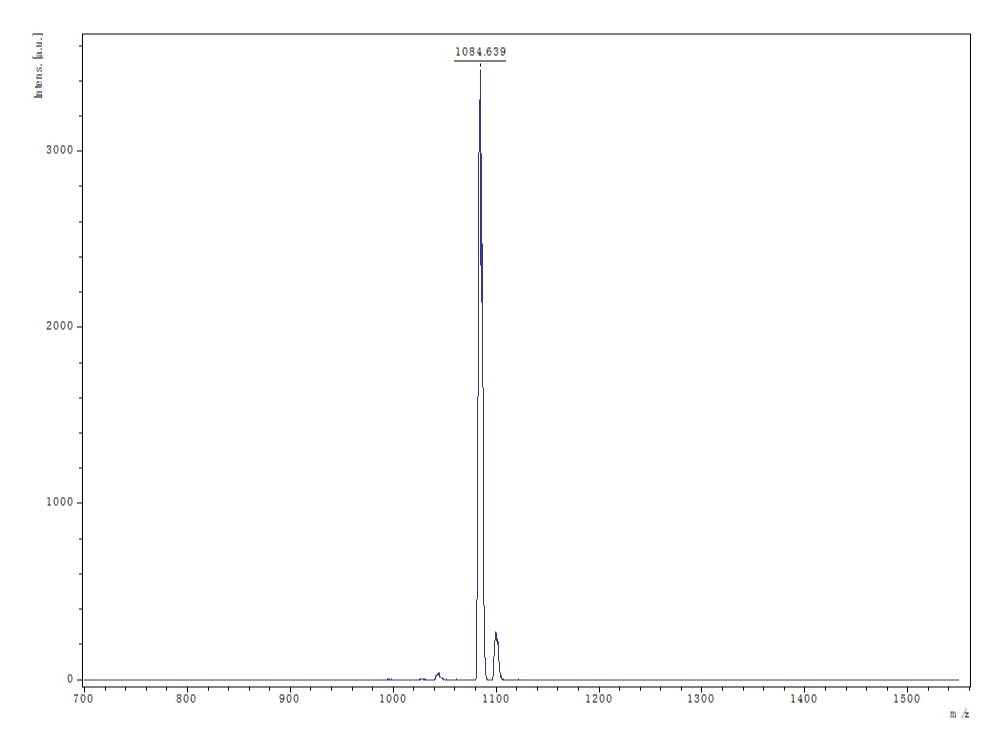


Supplementary Figure S6. TOF-MALDI-MS of tetra(2,3-bis(butylthio)-maleonitrile)porphyrazine with Zinc (ZnPz(Sbu)_8_).


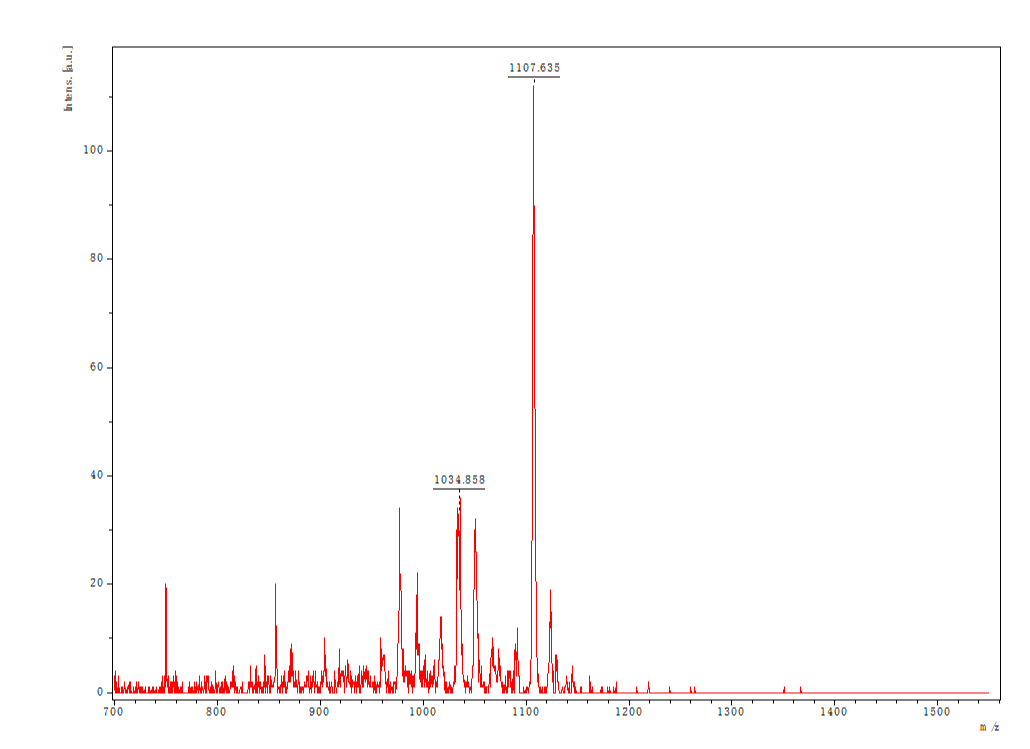


Supplementary Figure S7. TOF-MALDI-MS of tetra(2,3-bis(butylthio)-maleonitrile)porphyrazine with Manganese (MnPz(Sbu)_8_).


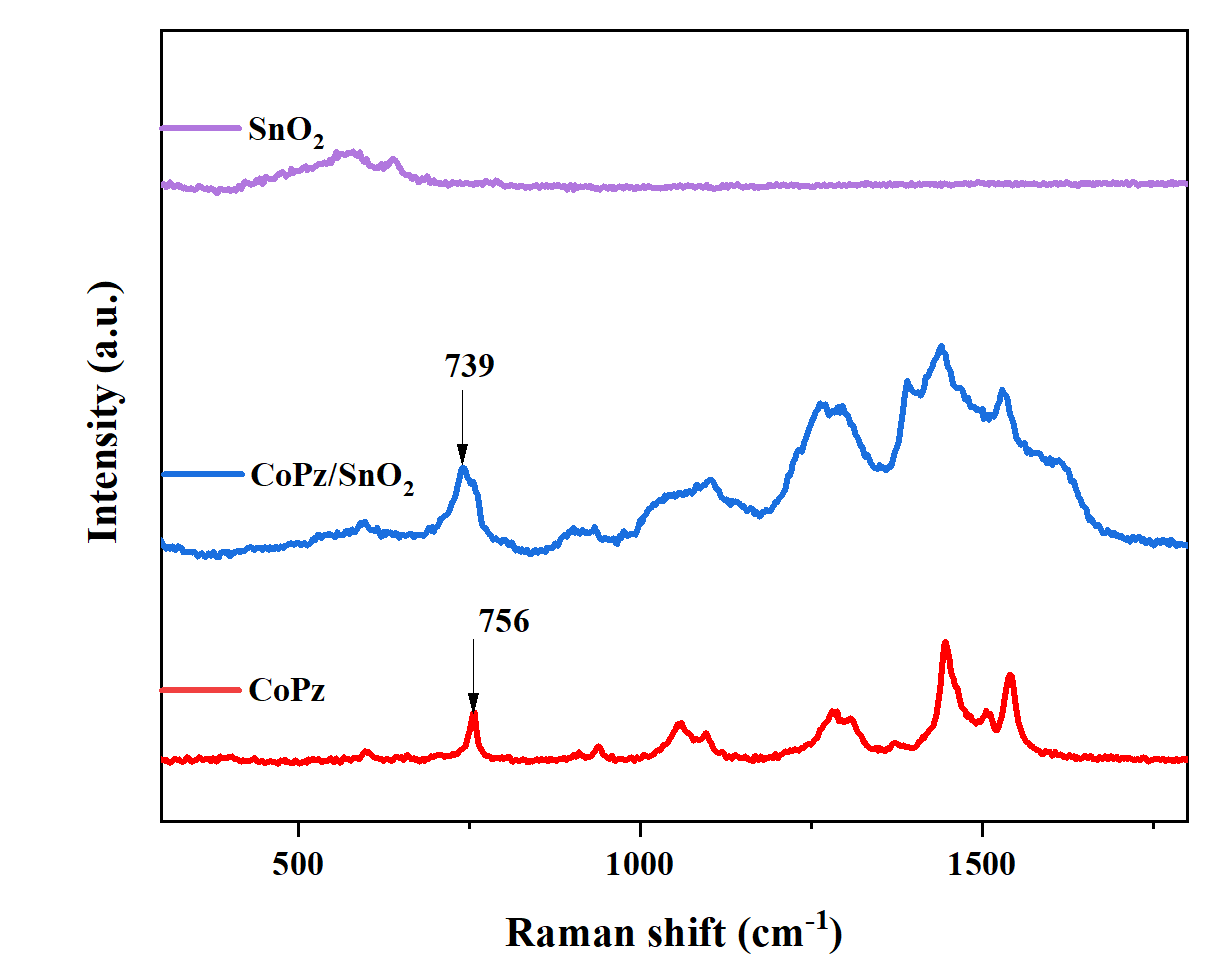


Supplementary Figure S8. Raman spectrum of CoPz, pure SnO_2_ and CoPz/SnO_2_.


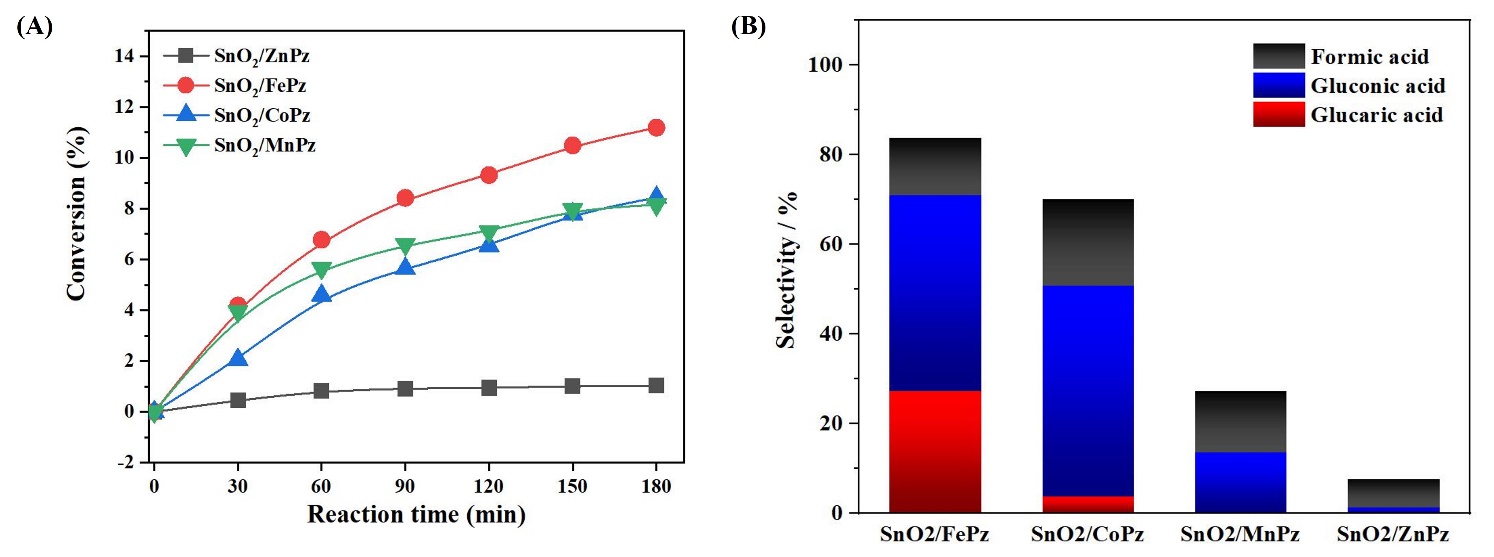


**Supplementary Figure S9.** The conversion of glucose (A) and the selectivity of organic acid (B) over SnO_2_/MPz composite under nitrogen atmosphere for 3h. Reaction conditions: aqueous glucose (1 mmol·L^-1^, 30 mL), catalyst (20 mg), light intensity (1.5 W·cm^-2^).
